# *In vitro* re-hardening of artificial enamel caries lesions using enamel matrix proteins or self-assembling peptides

**DOI:** 10.1590/1678-775720150352

**Published:** 2016

**Authors:** Patrick Schmidlin, Katja Zobrist, Thomas Attin, Florian Wegehaupt

**Affiliations:** University of Zurich, Center of Dental Medicine, Clinic of Preventive Dentistry, Periodontology and Cariology, Zurich, Switzerland.

**Keywords:** Tooth remineralization, Enamel, Hardness

## Abstract

**Objectives:**

To assess the re-hardening potential of enamel matrix derivatives (EMD) and self-assembling peptides *in vitro*, hypothesizing that these materials may increase the mineralization of artificial carious lesions and improve hardness profiles.

**Material and Methods:**

Forty-eight enamel samples were prepared from extracted bovine lower central incisors. After embedding and polishing, nail varnish was applied, leaving a defined test area. One third of this area was covered with a flowable composite (non-demineralized control). The remaining area was demineralized in an acidic buffer solution for 18 d to simulate a carious lesion. Half the demineralized area was then covered with composite (demineralized control), while the last third was left open for three test and one control treatments: (A) Application of enamel-matrix proteins (EMD - lyophilized protein fractions dissolved in acetic acid, Straumann), (B) self-assembling peptides (SAP, Curodont), or (C) amine fluoride solution (Am-F, GABA) for 5 min each. Untreated samples (D) served as control. After treatment, samples were immersed in artificial saliva for four weeks (remineralization phase) and microhardness (Knoop) depth profiles (25-300 µm) were obtained at sections. Two-way ANOVA was calculated to determine differences between the areas (re-hardening or softening).

**Results:**

Decalcification resulted in significant softening of the subsurface enamel in all groups (A-D). A significant re-hardening up to 125 µm was observed in the EMD and SAP groups.

**Conclusions:**

This study showed that EMD and SAP were able to improve the hardness profiles when applied to deep demineralized artificial lesions. However, further research is needed to verify and improve this observed effect.

## INTRODUCTION

Mineralization processes are based on complex interactions among organic and inorganic components in an aqueous fluid, which gradually transform the matrix from a protein-rich substance into hard and durable tissues^7^ ^,^ ^17^. Different enamel matrix proteins are involved in the mineralization processes of enamel, dentin, and cementum^23^. In the enamel matrix, 90% of the proteins belong to the amelogenin group, whereas the remaining 10% are prolin-rich non-amelogenins, tuftelin, or other serum proteins^2^ ^,^ ^10^. Nascent amelogenins are bipolar, with a hydrophilic carboxyl terminus, but are hydrophobic over most of their lengths^21^. Excreted from ameloblasts, amelogenins extracellularly assemble into nanospheres of approximately 20 nm and form a supramolecular structural framework^6^. *In vivo* and *in vitro* investigations have shown some putative functions of amelogenins^18^. They inhibit apatite nucleation^1^, but appear to direct crystal growth almost exclusively in the c-axis direction, controlling crystal orientation and texture formation. In this context it must be acknowledged, however, that the enamel proteins differ from the more acidic and phosphorylated matrix macromolecules that control biomineralization in bone and dentine^8^ ^,^ ^20^.

As a commercially available product, Emdogain^®^, comprising enamel matrix proteins, has attracted much attention in the past years^9^. However, few attempts have been made to evaluate the potential of this product and its protein constituents in mineralization processes of other dental hard tissues, namely enamel and dentin, and potential therapeutic applications, i.e., the remineralization potential of caries. Electrolytic deposition was examined in a very experimental *in vitro* set-up, and the formation of organized bundles in amelogenin-apatite composites was found to be mainly driven by amelogenin nanochain assembly, which already highlighted the potential of such applications in Dentistry^5^. A recombinant amelogenin rP172 also modulated apatite crystals, which resembled those formed in the early stage of tooth enamel biomineralization, suggesting the functional roles of amelogenins during the oriented growth of enamel crystallites and a great potential for amelogenins in applications designed to fabricate enamel-like calcium phosphate biomaterials^24^. However, to date, no straightforward studies were undertaken, according to the authors’ knowledge, to study the application of Emdogain^®^ when directly applied on demineralized enamel. On the other hand, other smart nanobiomaterials have been introduced in regenerative dentistry aiming to repair enamel lesions and self-assembling peptides, which may undergo spontaneous assembling after being applied to and infiltrating a carious lesion^11^. These substances are imitating “natural histogenesis” and introducing a fibrillar network, as previously mentioned, and they have been shown to lead to mineral deposition in the enamel lesion^13^.

This study aimed to assess the re-hardening potential of enamel matrix derivatives and self-assembling peptides *in vitro*, hypothesizing that these substances may improve the physico-mechanical properties of artificial enamel caries lesions by improving the hardness profile when directly compared to untreated demineralized enamel.

## MATERIAL AND METHODS

### Sample preparation

Twenty-four freshly extracted bovine lower incisors were used in this study. Roots were removed at the cementoenamel junction with a water-cooled diamond disc. Additionally, crowns were cut in half in the corono-incisal direction, resulting in a total of 48 enamel/dentine blocks, which were later embedded in acrylic resin (Paladur, Heraeus Kulzer, Hanau, Germany). The enamel surface was ground flat and polished using water-cooled silicon carbide paper (Stuers, Erkrat, Germany) up to P4000 grit, until an experimental window of approximately 5x8 mm available for this study. Samples were stored in water until needed for testing.

One third of the exposed enamel surface was left untreated and covered with a flowable composite resin (Orbi Flow, Lot N20538, ORBIS DENTAL, Münster, Germany) to serve as a non-demineralized control site for subsurface hardness measurements. After covering the non-demineralized control sites, each sample was subsequently demineralized in 20 mL agitated Buskes demineralization acidic buffer solution at 37°C for 18 days^4^. The demineralization acidic buffer solution was prepared by mixing 5 L distilled water, 2.205 g CaCl_2_+2H_2_O (3.0 mM), 2.041 g KH_2_PO_4_ (3.0 mM), 10 mL MHDP-solution (6 µm; prepared from 100 mL distilled water with 0.0528 g methylenediphosphoric acid), 14.3 mL CH_3_COOH (50 mM), and 10 M KOH to titrate the solution at pH 4.95. At every second day, the solution was refreshed. All chemical products used were purchased from Merck (VWR International AG, 8953 Dietikon, Switzerland). After the demineralization process, white-spot formation was visually assessed and samples were equally distributed, concerning caries formation, to four groups (A–D), by two investigators. After distribution of the samples, the middle area of the experimental window was also covered with the flowable composite material. This area served as the demineralized control site for hardness profiling. The last third of the experimental window was left uncovered, and either one of the three tests (A-C) and a control treatment (D) was applied as follows, respectively: (A) Application of enamel-matrix proteins/derivatives (EMD), i.e., lyophilized protein fractions dissolved in acetic acid Emdogain^®^ (Straumann, Basel, Switzerland; Lot: EMD 632 A Lot: EMD 632 A), (B) self-assembling peptides (SAP, Curodont Repair^TM^, Credentis AG, Windisch, Switzerland; Lot: 1006198), or (C) amine fluoride solution (GABA, Therwil, Switzerland; Lot 225612). Samples in group (D) were not treated, and were only stored in artificial saliva.

### Sample treatment

The following materials/solutions were prepared and used for the experiments:

- (A) Enamel matrix derivatives (EMD):

Lyophilized EMD was dissolved in 1 mL 0.05 M acetic acid and stored for 30 min at 4°C to a final concentration of 30 mg/mL and a pH of 3.5.

- (B) Self-assembling peptides (SAP):

The material was delivered in powder form, in glass vials, by the company. Just before application, 50 µL H_2_O was added. The solution was a prototype of the now commercially available product CURODONT Repair^TM^.

- (C) Amine fluoride solution (Fluoride):

The solution (Elmex-Fluid; 9250 ppm Olaflur and 750 ppm Dectaflur, GABA, Therwil, Switzerland) was used as delivered by the manufacturer.

From all solutions, 50 µL were applied on the respective samples and left for 5 min on the enamel surface. After application, the solution was carefully removed using a towel. Samples were then immediately stored in 20 mL artificial saliva per sample for 21 d at 37°C. The artificial saliva used was prepared by mixing 2.4 g KCl, 1.7 g NaCl, 0.1 g MgCl_2_^**.**^6H_2_O, 0.2 g CaCl_2_^**.**^2 H_2_O, 0.2 g KSCN, 0.7 g KH_2_PO_4_, and 0.1 g H_3_BO_3_ with distilled water to an end volume of 1 L. Each sample was separately stored and the storage media was changed every day.

### Hardness measurement

After treatment, samples were perpendicularly sectioned in such a way that all three areas could be measured. The sectioned surface was ground flat and polished using water-cooled silicon carbide paper (Stuers, Erkrat, Germany) up to P4000 grit. Subsurface microhardness (KHN) of the enamel samples was determined using 15 indentations (load weight 50 g, indentation time 20 s). The latter were made on the enamel surface of the samples using a Knoop hardness-measuring device (High Quality Hardness Tester, Buehler, Düsseldorf, Germany). Indentations started 25 µm below the enamel surface and were adjusted to minimum 50 µm from each other, with an offset of 50 µm in the lateral aspect, which resulted in an actual measurement of two lines. Thereby, depth was measured from 25 µm below the enamel surface to a depth of 300 µm.

### Statistical analyses

Descriptive statistics, such as mean and standard deviation (SD), were calculated for each test group separately. Data were analyzed to verify the normality of distribution using Kolmogorov-Smirnov and Shapiro-Wilk Test. Two-way ANOVA, followed by the *post hoc* Scheffé Test, were conducted to detect significant differences between the demineralized control site and the treated test site. All statistical analyses were performed with IBM SPSS (Version 2; IBM Armonk, NY, USA), and the significance level of p<0.05 was applied.

## RESULTS

The KHN values in the respective groups at respective depth levels are presented in Table 1.

**Table 1:** Mean (SD) Subsurface microhardness (KHN) values after treatment (treatment site) with the respective products (EMD, SAP, Fluoride, and saliva only) at the different depth levels after demineralization as compared to the demineralization (demin) sites without respective treatment. Significant differences in the KHN values between treatment and demin sites are marked with an asterisk (read horizontally)

	Treatment											
	EMD			SAP			Fluoride			Saliva only (control)		
Depth [µm]	Treatment site		Demin site	Treatment site		Demin site	Treatment site		Demin site	Treatment site		Demin site
25	81 (65)	*	54 (49)	52 (26)	*	43 (18)	75 (46)	*	62 (40)	73 (41)	*	55 (28)
50	110 (98)		84 (86)	85 (74)	*	57 (40)	101 (65)		102 (77)	100 (58)	*	84 (51)
75	155 (103)	*	110 (102)	143 (97)	*	94 (66)	148 (92)		145 (85)	144 (66)		130 (53)
100	193 (96)	*	144 (102)	195 (92)	*	149 (83)	182 (90)		187 (102)	220 (71)		195 (56)
125	247 (63)	*	196 (89)	258 (66)	*	200 (90)	225 (85)		225 (82)	273 (49)		250 (28)
150	279 (49)	*	245 (68)	288 (51)	*	245 (65)	254 (71)		254 (78)	290 (43)		292 (27)
175	306 (29)	*	276 (60)	321 (28)	*	280 (46)	285 (58)		287 (51)	305 (24)		313 (27)
200	324 (21)	*	292 (15)	326 (22)	*	306 (29)	297 (52)		313 (33)	319 (25)		324 (18)
225	328 (15)		305 (40)	329 (19)		317 (21)	311 (31)		313 (30)	333 (18)		331 (18)
250	329 (17)		312 (38)	333 (23)		326 (20)	317 (26)		321 (22)	335 (15)		332 (18)
275	333 (15)		322 (25)	335 (19)		330 (14)	326 (18)		326 (16)	336 (18)		334 (18)
300	333 (22)		328 (16)	334 (15)		332 (14)	329 (15)		328 (19)	332 (19)		333 (19)

The hardness values of the non-demineralized control site ranged from 282 KHN to 344 KHN and showed no significant difference between the different groups (data not shown).

The lowest hardness values were detected in the subsurface zone, i.e., 25 µm below the enamel surface of the demineralized control site, and ranged from 43±18 KHN to 62±40 KHN. Since these results indicate differences in the softening of the enamel in the different groups, the hardness values were later compared only within the same group between the demineralized control site and the treated test site to indicate up to which depth the respective treatments were able to induce re-hardening.

Statistically significant differences in KHN between the demineralized control site and the treated test site were observed in the first 25 µm in all groups. A significant re-hardening in deeper zones was detected in groups A (treated with EMD) and B (treated with SAP) in regions up to 200 µm (p≤0.05, respectively).

In no group, significantly lower KHN values were observed for the treatment site when compared to the respective demineralized sites, indicating no softening due to the application of the respective products.

## DISCUSSION

This study assessed the re-hardening potential of enamel matrix derivatives and self-assembling peptides *in vitro* when focusing on subsurface hardness profiles of artificial carious lesions.

For the study, samples were prepared from bovine lower incisors, although bovine and human enamel are not identical due to genetic, environmental, and dietary differences^14^. However, bovine teeth are easier to obtain in large quantities compared with human teeth^19^. Additionally, bovine teeth are in better condition and with a more uniform composition^25^ when extracted for study purposes. Another reason for the use of bovine teeth is their larger size, which allows the preparation of more samples from the same tooth, resulting in a reduction in differences between the samples^14^. Artificial caries lesions are formed faster in bovine enamel than in human enamel, although the resulting lesions were almost indistinguishable in their mineral distribution characteristics^15^. Since the data for the re-hardening were only compared within the same sample (between the demineralized control site and the treated test site) and will only be interpreted within the different groups of the present study (relative data), we assume that the advantages of using bovine teeth will outbalance the disadvantages of not using human teeth.

Significant differences in KHN between untreated demineralized and treated sites, indicating a re-hardening deeper than 50 µm – as hypothesized - were only found in samples on which EMD or SAP was applied. However the effects, despite being statistically significant, should still be considered with caution. In general, the differences were rather small. Although comparative data from microradiography and micro-hardness measurements have shown some correlation^6^ ^,^ ^12^, a recent study by Magalhães, et al.^16^ (2009) concluded that cross-sectional hardness measurement, used as an alternative to transverse microradiography (TMR), is not very accurate for estimating the mineral content, but it gives some information regarding the mechanical (physical strength) properties of the lesions, which are not provided by TMR^16^. In this context, we should mention that the starting point of our measurements was set at 25 μm below the enamel surface, whereas other studies used to measure from 10 μm below the surface. However, we observed in preliminary tests that the uncovered enamel surface would break when starting at 10 μm below the surface, since the samples were too fragile due to the severe demineralization pattern of our samples. Only when setting the first indentation at 25 μm we were able to achieve adequate readings for all groups. The starting point, therefore, relies on the experimental set-up and the demineralization conditions: whereas some studies started 10 µm from the surface after a demineralization time of 6 d on enamel^3^, in another study, one could only start 50 µm from the cavity floor, when dentin was used as a substrate and a demineralization period of only 2 h was chosen^22^. In addition, an ultramicroindentation system in the latter studies was used, which also influences the selection of the starting points.

Therefore, one should be careful when interpreting the findings of the present study (re-hardening due to application of EMD or SAP) as remineralization. In contrast, Kirkham, et al.^13^ (2007) detected a net mineral gain in artificial white spot lesions after an application of a SAP and remineralization in a pH-cycling model for five days^13^.

Considering these results and the findings of the present study, one might conclude that the approach is promising and deserves further investigation.

Recently, a press-release was published in the Swiss Journal of Dentistry, which reported on a round table discussion on so-called biological enamel regeneration, which defined the therapy with SAP products (analogous to guided bone or tissue regeneration) as “Guided Enamel Regeneration”. However, for the reasons stated above, one should be careful when using the term “enamel regeneration”, as even a remineralization, as found by Kirkham, et al.^13^ (2007), does not necessarily imply enamel regeneration. Calling an uptake of minerals in the caries lesion “enamel regeneration” might be an overstatement, since for enamel regeneration to take place there must be not only mineral uptake, but also a crystallization of these minerals in terms of apatite formation^13^.

Furthermore, no information is available as to whether enamel regeneration is really necessary. One might assume that a re-hardening of carious lesions is satisfactory, as this will result in an increase in the mechanical stability of the lesion, thereby preventing a breakdown of the lesion surface and the formation of a cavity. To date, one can only speculate about the chemical and structural re-organization in the newly formed subsurface zone and whether a fibrillar network has been established, which consists of amelogenin- or fibril-apatite composites, as it as been found in earlier studies^11^ ^,^ ^13^ ^,^ ^24^.

But within the limitation of the present study, it might be concluded that the application of enamel matrix derivatives and self-assembling peptides on artificial carious lesions can improve the re-hardening of these lesions, also in deeper layers of the lesion. Further studies are needed to verify this approach, also under pH-cycling conditions and *in vivo*.

## CONCLUSION

Within the limitations of this study, EMD and SAP were able to improve the hardness profiles applied to deep demineralized artificial lesions.
